# Supplementary material for: Differential Expression of Stress Adaptation Genes in a Diatom Ulnaria acus under Different Culture Conditions
Source: Int J Mol Sci. 2024 Feb 15;25(4):2314. doi: 10.3390/ijms25042314 (PMC10888605; doi:10.3390/ijms25042314)
Supplement: Supplementary file 1 [file ijms-25-02314-s001.zip › Supplement Figure S1.pdf]

Supplementary Figure S1. Putative DSP phosphorylation sites in *Thalassiosira pseudonana* (TpDSP1), *Phaeodactylum tricornutum* (PtDSP) and *Ulnaria acus* (UaDSP). *In silico* prediction was performed using NetPhos 2.0. Phosphorylation sites are highlighted in gray.

```
        ....|....|....|....|....|....|....|....|....|....|....|....|....|....|....|....|....|....|
            5        15        25        35        45        55        65        75        85
TpDSP1  MIAQKKALLATLTIVALNNANAFVVPSTTTARPFISRPLYESSMVDQQESTTSYQQQHDDDDN---DTNKLIMDIPPTPKAT--------
PtDSP   MAKLTSIALCAMLTSSSAFTPTFSSPKQYTCLRMSDLPRQAIPDSIYMQIAMEIPSKNGHTATGFPNLQEQVIPQQVHPVESDRVSLSAQ
UaDSP   -MNTYYLLLATLLGTEVK---AFIVQPISGCRLQMS--VASSSNHHFMEEAVSIIDESKAQVT---EKNE--MPQAAKPKVS--------

        ....|....|....|....|....|....|....|....|....|....|....|....|....|....|....|....|....|....|
             95       105       115       125       135       145       155       165       175
TpDSP1  ----------PTPKNPAHKEGIFSPLVYAASTVIGPEQLNKVRAQIISLHSDIIKSFVSTSDSTLGKAILRQLFEMTDADNSGYLDKREV
PtDSP   ALRQKSKPTLPKKMTAKHGDGLFSPLVKLFKAALGDDRLNKIRAKAIATHSEVIASFVETAESASGEAVLDTLFALSDKNGDGHIDEGEL
UaDSP   ----------KQAKS-HGKDGLLSPIVTSLKAVIGDDELNKLRGKVIAMHSDIIKSFVDTADTRLGQAVLKMMFDVADVDGSGTVERDEL

        ....|....|....|....|....|....|....|....|....|....|....|....|....
           185       195       205       215       225       235
TpDSP1  EAALNLLGFKWLKEKHVEKIFERADLNSDGEISLEEFMAEAPKTLKVNLVKLAKNNGGDMGLLV
PtDSP   KEALRTLGFAWIEEKQAKGILSRADKDKKGYITKDEWKAEAPKTLRTNLTKLAKKNGGDLGFLV
UaDSP   ERSLKALGFRFLNEKQIKGIFDRADLDKDGHIDLEEWLTEAPKTLRTNLVKLAKQNGGDMGLLA
```
