# Supplementary material for: Differential Expression of Stress Adaptation Genes in a Diatom Ulnaria acus under Different Culture Conditions
Source: Int J Mol Sci. 2024 Feb 15;25(4):2314. doi: 10.3390/ijms25042314 (PMC10888605; doi:10.3390/ijms25042314)

Supplementary Figure S2. Changes in transcription levels of the studied genes (according to Galachyants et al., 2019). A is the transfer of cells from the exponential growth phase (exp) to darkness (dark). B – the return of cells from darkness to light. * - statistically significant decrease in the level of transcription (p<0.05).

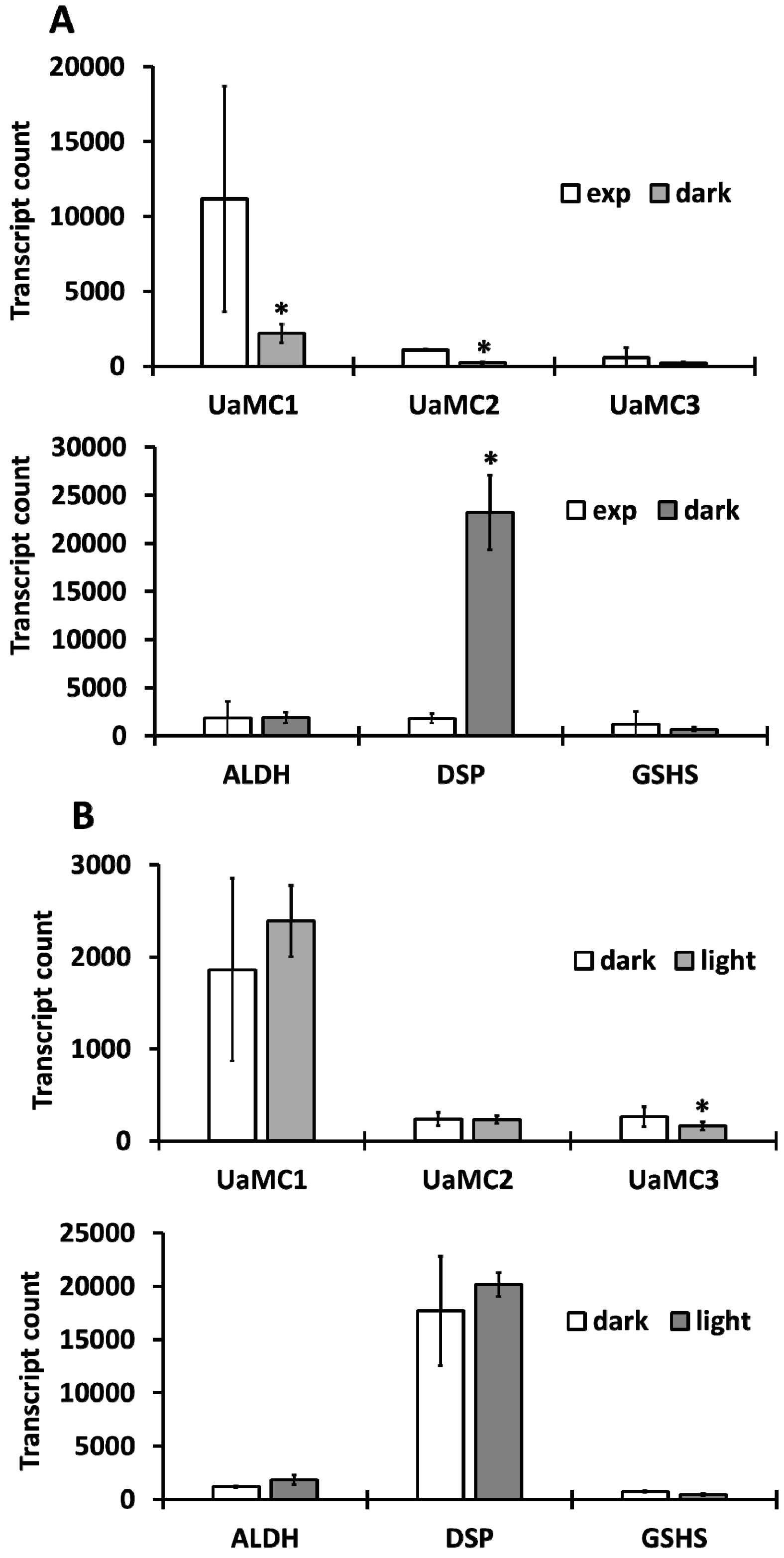

Supplement: Supplementary file 1 [file ijms-25-02314-s001.zip › Supplement Figure S2.pdf]
