# Supplementary material for: Differential Expression of Stress Adaptation Genes in a Diatom Ulnaria acus under Different Culture Conditions
Source: Int J Mol Sci. 2024 Feb 15;25(4):2314. doi: 10.3390/ijms25042314 (PMC10888605; doi:10.3390/ijms25042314)
Supplement: Supplementary file 1 [file ijms-25-02314-s001.zip › Supplement Table S1.pdf]

Supplementary Table S1. Primers for amplification of metacaspase genes. The name indicates the name of the contigue, as well as the position of the first nucleotide of the primer in it.

Primer	**Sequence (5’→3’)**
14822_metcasp_663F	GAACAATGGCACACATTTCTCCTGGT
14822_metcasp_1309F	ACTCTCTTCCAGCACTCCTATTGATG
14822_metacasp_1915R	CCTCCGACTTTTCCAAA
18835_metcasp_1848F	CTGTCAATCGCCTCTTCACTCAT
18835_metcasp_2375F	TTCTTCCTTGTCTCTCACTCTCACAC
18835_metcasp_3619R	AGGTTTTGTTTTGATCCATCC
10107_6F	ATCGTGATCGGCGATGTAAC
10107_604F	CTCAATATCACCAATTTCCCG
10107_1712R	TCAAGCTGTATTCCGCGCTA
7884_metcasp_2F	CTTGATTGACGTATCCAGCTACTA
7884_metcasp_1443R	TGTAGGGCATTCGCTGACTCT
12375_metcasp_1F	CTCTCCCACTACTTTTTTTCTAGT
12375_metcasp_1443R	ATTGTAGGGCATTCGCTGACT
23175_metcasp_66F	ACGTCAGCTCGCCATGATTAAAATA
23175_metcasp_621F	TCCTCCTATGTGTTGGATGATGA
23175_metcasp_1223F	TCAAGCCCCGACACTCAC
23175_metcasp_2476R	AAACAACGTGGAATCTTTAACTAGAA
22059_metcasp_629F	TTTTGGATGA TGAAGTGAAA GTTA
22059_metcasp_1219F	TTC GCTCAAGCGC CGACACTC
22059_metcasp_2476R	AAACAACGTGGAATCTTTTACTAGAA
5582_metcasp_3F	GGTCAAGTTGTTACAATTTGAAAT
5582_metcasp_1607R	CCTCTAAATACGTTAACCATTGT
