# Supplementary material for: Differential Expression of Stress Adaptation Genes in a Diatom Ulnaria acus under Different Culture Conditions
Source: Int J Mol Sci. 2024 Feb 15;25(4):2314. doi: 10.3390/ijms25042314 (PMC10888605; doi:10.3390/ijms25042314)
Supplement: Supplementary file 1 [file ijms-25-02314-s001.zip › Supplement Table S2.pdf]

Supplementary Table S2. Characteristics of U. acus metacaspase genes.

Contig number * of *U. acus* genome sequence	Position of the MC gene in the contig	Abbreviation in this paper	Abbreviation according to Morozov, 2019	Accession number of the ORF in NCBI	Gene size, b.p.	ORF length, b.p.	Position of introns in the gene
14822	1001-1957	*UaMC1*	Sacus5	OR231937	957	957	absence
18835	671-1919	*UaMC2*	Sacus6	OR231939	1249	921	82-335; 845-920
10107	90-1370	*UaMC3*	Sacus3	OR231938	1281	1281	absence
22059	201-2306	*UaMC4*	Sacus7	OR231943	2106	2106	absence
23175	201-2306	*UaMC5*	Sacus8	OR231944	2106	2106	absence
7784	201-1243	*UaMC6*	Sacus2	OR231945	1043	963	746-826
5582	176-1218	*UaMC7*	Sacus1	OR231946	1043	963	746-826
12375	201-1243	*UaMC8*	Sacus4	OR231947	1043	963	746-826

* - by date Galachyants et al., 2015, Assembly and annotation data for the U. acus complete genome are freely available at http:// lin.irk.ru/sacus
