# Supplementary material for: Differential Expression of Stress Adaptation Genes in a Diatom Ulnaria acus under Different Culture Conditions
Source: Int J Mol Sci. 2024 Feb 15;25(4):2314. doi: 10.3390/ijms25042314 (PMC10888605; doi:10.3390/ijms25042314)
Supplement: Supplementary file 1 [file ijms-25-02314-s001.zip › Supplement Table S3.pdf]

Supplementary Table S3. Features of predicted DSP amino acid sequences.

Species	NCBI accession number	Abbreviation	Length, a.a.	EF-hand domains	Signal peptide	Prediction of transmembrane helices in protein
Thalassiosira pseudonana	ABU86411.1	TpDSP1	223	138-166<br>175-203	1-22	7-29
	ABU86412.1	TpDSP2	230	145-173<br>182-210	1-24	-
Skeletonema costatum	AAY27742.1	ScDSP1	232	147-175<br>184-212	1-23	7-29
	ABU86410.1	ScDSP2	248	159-186<br>194-222	1-23	7-25
Chrysochromulina tobinii	KOO53112.1	CtDSP1	243	158-186<br>195-223	-	-
	KOO24320.1	CtDSP2	243	158-186<br>195-223	1-20	-
Emiliania huxley	XP_005778932.1	EhDSP1	163	78-106<br>115-143	-	-
	XP_005773091.1	EhDSP2	154	74-102<br>111-139	-	-
Phaeodactylum tricornutum	ABU86413.1	PtDSP	244	159-187<br>196-224	1-18	-
Fragilariopsis cylindrus	OEU22427.1	FcDSP	187	102-130<br>139-167	-	-
Fistulifera solaris	GAX13799.1	FsDSP	210	124-152<br>162-190	1-25	7-28
Thalassiosira oceanica	EJK59087.1	ToDSP	362	277-305<br>314-342	-	-
Ulnaria acus	OR677822	UaDSP	214	129-157<br>166-194	1-18	-
