# Supplementary material for: Differential Expression of Stress Adaptation Genes in a Diatom Ulnaria acus under Different Culture Conditions
Source: Int J Mol Sci. 2024 Feb 15;25(4):2314. doi: 10.3390/ijms25042314 (PMC10888605; doi:10.3390/ijms25042314)
Supplement: Supplementary file 1 [file ijms-25-02314-s001.zip › Supplement Table S4.pdf]

Supplementary Table S4. ALDH12 amino acid sequences.

Species	NCBI accession number	Abbreviation	Length, a.a.
Arabidopsis thaliana	NP_568955.1	AtALDH12	556
Skeletonema marinoi	ANG60897.1	SmALDH12	544
Thalassiosira pseudonana	XP 002295441.1	TpALDH12	537
Fragilaria crotonensis	KAI2494282.1	FcALDH12A	553
	KAI2491558.1	FcALDH12B	586
Nitzschia inconspicua	KAG7363017.1	NiALDH12A	564
	KAG7341873.1	NiALDH12B	563
Phaeodactylum tricornutum	XP 002177243.1	PtALDH12	551
Chaetoceros tenuissimus	GFH52268.1	CtALDH12	552
Ulnaria acus	OR677820	UaALDH12	560
